# ‘Hearts and minds’: association, causation and implication of cognitive impairment in heart failure

**DOI:** 10.1186/s13195-015-0106-5

**Published:** 2015-02-27

**Authors:** Jane A Cannon, John JV McMurray, Terry J Quinn

**Affiliations:** British Heart Foundation Glasgow, Institute of Cardiovascular and Medical Sciences, University of Glasgow, University Avenue, Glasgow, G12 8TA UK; Department of Academic Geriatric Medicine, Institute of Cardiovascular and Medical Sciences, New Lister Building, Glasgow Royal Infirmary, Glasgow, UK G4 0SF UK

## Abstract

The clinical syndrome of heart failure is one of the leading causes of hospitalisation and mortality in older adults. An association between cognitive impairment and heart failure is well described but our understanding of the relationship between the two conditions remains limited. In this review we provide a synthesis of available evidence, focussing on epidemiology, the potential pathogenesis, and treatment implications of cognitive decline in heart failure. Most evidence available relates to heart failure with reduced ejection fraction and the syndromes of chronic cognitive decline or dementia. These conditions are only part of a complex heart failure-cognition paradigm. Associations between cognition and heart failure with preserved ejection fraction and between acute delirium and heart failure also seem evident and where data are available we will discuss these syndromes. Many questions remain unanswered regarding heart failure and cognition. Much of the observational evidence on the association is confounded by study design, comorbidity and insensitive cognitive assessment tools. If a causal link exists, there are several potential pathophysiological explanations. Plausible underlying mechanisms relating to cerebral hypoperfusion or occult cerebrovascular disease have been described and it seems likely that these may coexist and exert synergistic effects. Despite the prevalence of the two conditions, when cognitive impairment coexists with heart failure there is no specific guidance on treatment. Institution of evidence-based heart failure therapies that reduce mortality and hospitalisations seems intuitive and there is no signal that these interventions have an adverse effect on cognition. However, cognitive impairment will present a further barrier to the often complex medication self-management that is required in contemporary heart failure treatment.

## Definitions and burden of heart failure

The term 'heart failure' (HF) is used to describe a condition wherein cardiac output is insufficient to meet metabolic requirements [1]. Clinically, it is defined as a syndrome where patients have typical signs and symptoms resulting from an abnormality of cardiac structure or function [2]. Contemporary terminology used to describe HF is based on left ventricular ejection fraction (EF). This is considered important not only because of prognosis (the lower the EF the poorer the survival) but also because the major trials that inform the evidence base have almost exclusively focussed on patients who have HF with reduced ejection fraction (HF-REF) [2]. A subgroup of patients also present with classical signs and symptoms but in the context of preserved ejection fraction (HF-PEF). These patients often have evidence of diastolic dysfunction and this is considered by many as the cause of HF symptoms.

It is estimated that 1 to 2% of the adult population in developed countries have HF with the prevalence increasing to ≥10% among patients aged over 70 years; more than half of these patients have HF-REF [3]. The most common underlying aetiology in HF-REF is coronary artery disease (CAD) resulting in myocardial damage. Other common causes include hypertension, valvular pathology, viral infection and alcohol excess [2]. HF-PEF is more common in older, female patients. It is less frequently due to CAD and more often linked to hypertension and atrial fibrillation (AF), with the diagnosis being one of exclusion of other non-cardiac causes of breathlessness [2].

**Figure 1:**
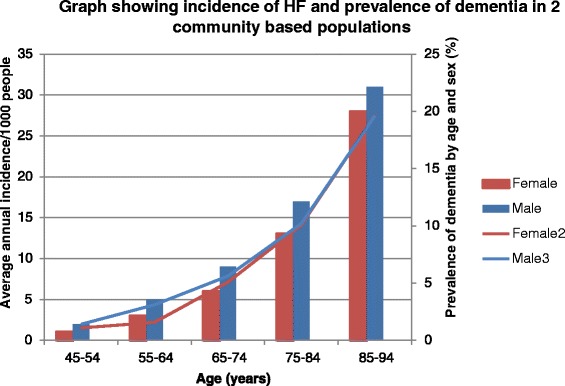
**Incidence of heart failure within the Framingham cohort and prevalence of dementia by age and sex (pooled from five centres of the Medical Research Council cognitive function and ageing study)**. Authors’ own figure based on data from [5]. HF, heart failure.

HF admissions account for 5% of all medical admissions (making it the commonest cause of unscheduled admission in older adults) and 2% of the total UK National Health Service budget [4]. Societal and demographic changes, including aging of the general population and improved survival from CAD, will increase HF prevalence (Figure 1) with a potential doubling in HF prevalence within the next 40 years [2].

## Heart failure and cognitive impairment – strength of association

The co-existence of symptomatic 'heart failure' and 'brain failure' has been recognised for decades, with a description of 'cardiogenic dementia' first introduced in the 1970s. While the co-occurrence of HF and cognitive problems will be familiar to most clinicians, the topic has received relatively little research interest compared with other aspects of cardiac disease. In collating and offering a synthesis of the available literature describing the association of HF and cognition, we have found a disparate and inconsistent literature, characterised by small sample sizes, heterogeneity and multiple potential biases. We provide a brief narrative overview of the field and have tabulated a more detailed summary of findings from available cross-sectional and prospective studies (Tables 1 to 3).

**Table 1:** **Studies examining the prevalence of cognitive impairment in patients with heart failure**

**Study**	**Sample**	**Population**	**Median age in years (SD)**	**Study methodology**	**Inclusion criteria**	**Exclusion criteria**	**CV measures/criteria**	**Cognitive assessment tool(s) used**	**Results**
Zuccalà 1997 [6]	57 HF pts	Consecutive admissions to hospital	77	Cross-sectional	Not specified	Co-morbid psychiatric or physical illness and previous diagnosis of CI	LVEF (mean EF 45%) NYHA II-III	MMSE, MDBandRCPM	53% of HF pts showed global CI with MMSE less than 24
Callegari 2002 [7]	64 HF pts, 321 healthy controls	Age <65 years and consecutive admissions to hospital	52 (8)	Cross-sectional	Not specified	Co-morbid psychiatric or neurological illness. Previous diagnosis of CI and female sex	LVEF <50%	Multidomain neuropsychiatric battery	HF pts scored lower than control group in short-term verbal memory, short-term visuospatial memory and visual spatial logical ability
							NYHA I-III		
							Cardiopulmonary testing with treadmill		
							Right heart catheterisation		
Trojano 2003 [8]	149 HF NYHA II pts	Age >65 years and consecutive admissions to hospital	HF NYHA II: 75 (7)	Cross-sectional	Not specified	Co-morbid psychiatric, neurological or physical illness. Previous diagnosis of CI	No measure of LV function	Multidomain neuropsychiatric battery	HF pts scored worse than those without HF in domains of: attention, verbal fluency, verbal learning
	159 HF NYHA III/IV		HF NYHA III/IV: 77 (7)				NYHA II-IV		No significant difference between pts with moderate or severe HF
	207 non-HF controls		Non-HF controls: 74 (7)						
Zuccalà 2005 [9]	1,511 HF pts, 11,790 control patients	All geriatric or general medical hospital admissions	79 (9)	Cross-sectional	Not specified	Not specified	HF diagnosis based on discharge documentation	Hodkinson abbreviated mental test	35% of HF pts showed global CI 29% of non-HF pts showed global CI
Feola 2007 [10]	60 HF-REF	Consecutive admissions to hospital	66	Cross-sectional	HF-REF: clinical HF, NYHA II-IV, LVEF ≤50%	Not specified	LVEF	Multidomain neuropsychiatric battery	23% of HF pts showed global CI
	12 HF-PEF				HF-PEF: diagnosed based on E/A ratio, deceleration time and LV dilatation		NYHA II-IV		
							BNP		
Debette 2007 [11]	83 HF pts	Consecutive admissions to hospital	62	Cross-sectional	Not specified	Hearing/visual impairment	LVEF <45%	MMSE	61% of HF pts showed global CI
							NYHA I-IV		
Dodson 2013 [12]	282 decompensated HF pts	Age >65 years and non-consecutive admissions to hospital	80 (8)	Prospective	English speaking	Co-morbid psychiatric illness	HF diagnosis based on documentation in medical records	MMSE	25% of HF pts showed evidence of mild CI 22% of HF pts showed moderate to severe CI
Schmidt 1991 [13]	20 iDCM pts	Age <50 years and ambulatory outpatients only	iDCM: 38 (5)	Cross-sectional	Not specified	Co-morbid psychiatric, neurological or physical illness	LVEF 14-45%	LGT-3 and ALID	Systolic HF pts performed worse than the control group in domains of attention, learning and memory and reaction time
	20 healthy controls		Healthy controls: 41 (8)				NYHA II-IV		
Grubb 2000 [14]	20 HF pts with CADs	Ambulatory outpatients only	HF: 68	Cross-sectional	Not specified	Co-morbid psychiatric or neurological illness. Previous hospital admission within 6 months	HF: LVEF <40%, NYHA III/IV	RBMT and WMS	No difference between HF pts and control group
	20 CAD control		CAD controls: 67				CAD controls: LVEF >55%, no CHF		
Riegel 2002 [15]	42 HF pts	Ambulatory outpatients only	75 (12)	Cross-sectional	English speaking	Co-morbid physical or psychiatric illness	No measure of LV function	MMSE and CIMS	29% of HF pts showed evidence of global CI
							NYHA I-IV		
Vogels 2007 [16]	62 HF pts	Age >50 years and ambulatory outpatients only	HF: 69 (9))	Case control	HF pts: diagnosis of HF >6 months and stable on medication >4 weeks	Co-morbid physical, neurological or psychiatric illness. Previous diagnosis of CI	LVEF <40%	Multidomain neuropsychiatric battery	HF pts scored lower than the healthy control group in all domains
	53 CAD controls		CAD controls: 69 (10)		CAD controls: IHD but no clinical CHF and EF >40%		NYHA II-IV		HF pts scored lower than the IHD control group in domains of memory and mental speed
	42 healthy controls		Healthy controls: 67 (9						IHD control group scored lower than the healthy control group in language only
Hoth 2008 [17]	31 HF pts	Age >55 years and ambulatory outpatients only	HF: 69 (9)	Cross-sectional	English speaking	Co-morbid physical, neurological or psychiatric illness. Previous diagnosis of CI	LVEF <40%	Multidomain neuropsychiatric battery	Systolic HF pts scored lower than the IHD control group in domains of executive function and cognitive flexibility
	31 CAD controls		CAD controls: 69 (9)		Minimum of 8th grade education		NYHA II-IV		
					CAD controls: angina/previous MI/PCI/PVD and HF excluded on basis of clinical examination				
Beer 2009 [18]	31 HF pts	Ambulatory outpatients only	HF: 54 (11)	Case control	Not specified	Co-morbid neurological illness or previous diagnosis of CI	LVEF <40%	Block design, CVLT and 'F,A,S test'	Systolic HF pts scored lower than control group in all cognitive domains
	24 healthy controls		Healthy controls: 56 (8)				NYHA II		
							LWHFQ		
Stanek 2009 [19]	40 HF pts, 35 CAD controls	Ambulatory outpatients only	70 (8)	Prospective	English speaking	Co-morbid psychiatric or neurological illness. Previous diagnosis of CI	NYHA II-III	DRS	No difference between systolic HF pts and CAD control patients in all domains
					CAD controls: history of MI, CAD, cardiac surgery, hypertension		CO <4 L/minute on echo		
Sauvé 2009 [20]	50 HF pts 50 healthy controls	Age >30 years in HF pts and >55 years in controls. Ambulatory outpatients only	HF: 63 (14) Healthy controls: 63 (14)	Case control	Diagnosis of HF >6 months	Co-morbid psychiatric or neurological illness	LVEF ≤40% NYHA II-IV	Multidomain neuropsychiatric battery	Systolic HF pts scored lower than control group in domain of verbal memory
Pressler 2010 [21]	249 HF pts	Ambulatory outpatients only	HF: 63 (15)	Cross-sectional	HF: LVEF ≤40% and clinical HF	Co-morbid psychiatric, neurological or physical illness. Previous diagnosis of CI	NYHA	Multidomain neuropsychiatric battery	HF group performed worse than healthy and general medical groups in domains of memory, executive function and psychomotor speed
	63 healthy controls		Healthy controls: 53 (17)		Healthy controls: absence of any medical condition or controlled CV risk factors		LVEF		
	102 general medical pts		Medical group: 63 (12)		Medical group: major chronic disorder other than HF				
Bauer 2012 [22]	51 HF-REF, 29 HF-PEF	Age >21 years and ambulatory outpatients only	72 (12)	Cross-sectional	HF-REF: history of HF-REF >6 months, stable on medication >4 weeks, LVEF ≤40%	Co-morbid psychiatric, neurological or physical illness. Previous diagnosis of CI	LVEF	Multidomain neuropsychiatric battery	HF-REF and HF-PEF pts performed worse than age- and educated-adjusted healthy control groups in executive function, attention, language, memory and psychomotor speed
					HF-PEF: history of HF-PEF >6 months, stable on medication >4 weeks, LVEF >41%		NYHA		
Festa 2011 [23]	169 HF-REF, 38 HF-PEF	Age >17 years and ambulatory outpatients only	69	Retrospective	On medical treatment for HF	Co-morbid neurological illness	LVEF	Multidomain neuropsychiatric battery	Low EF was associated with poor memory in pts over 63 years old
					Haemodynamically stable				Pts <63 years old had preserved memory function regardless of EF.
					Not receiving mechanical circulatory support				
Steinberg 2011 [24]	55 HF pts	Ambulatory outpatients only	55 (8)	Cross-sectional	Stable clinical status	Co-morbid neurological or physical illness. Previous diagnosis of CI	LVEF ≤45%	Multidomain neuropsychiatric battery	44% of HF pts showed evidence of global CI
							NYHA I-III		
							6 minute walk test		
Jefferson 2011 [25]	1,114 pts from Framingham Heart Study	Age >40 and <89 years and ambulatory outpatients only	67 (9)	Cross-sectional	Not specified	Co-morbid neurological illness or previous diagnosis of CI	LVEF	Multidomain neuropsychiatric battery	U-shaped association between LVEF and cognitive performance
							Cardiac MRI		
Miller 2012 [26]	140 HF pts	Age >50 and <85 years and ambulatory outpatients only	69 (9)	Cross-sectional	English speaking	Co-morbid psychiatric or neurological illness	No measure of LV function	Multidomain neuropsychiatric battery	62% of HF pts showed evidence of global CI
							No NYHA classification		
							2 minute step test		
Almeida 2012 [27]	35 HF pts	Age >45 years and ambulatory outpatients only	HF: 69 (9)	Cross-sectional	HF: EF <40%, clinical HF ≥6 months, English speaking, NYHA I-III	Co-morbid psychiatric, neurological or physical illness. Previous diagnosis of CI	LVEF	Multidomain neuropsychiatric battery	HF pts scored lower than the healthy control group in domains of immediate/long-term memory and psychomotor speed
	56 CAD controls		CAD controls: 67 (10)		CAD controls: previous MI, English speaking, EF ≥60%, no clinical HF		NYHA		No difference between the HF group and IHD control group in cognition
	64 healthy controls		Healthy controls: 69 (11)		Healthy controls: English speaking, no previous MI/angina, EF ≥60%				
Hawkins 2012 [28]	251 HF pts	Ambulatory outpatients only	66 (10)	Cross-sectional	English speaking	Co-morbid psychiatric illness. Previous diagnosis of CI	LVEF ≤40%	Multidomain neuropsychiatric battery	58% of HF pts had CI with poor scores in the domains of verbal learning and verbal memory
Bratzke-Bauer 2013 [29]	47 HF-REF	Age >50 years and ambulatory outpatients only	HF-REF: 75 (9)	Cross-sectional	History of HF >6 months	Co-morbid psychiatric, neurological or physical illness. Previous diagnosis of CI	LVEF	Multidomain neuropsychiatric battery	23% of the HF-REF cohort showed evidence of CI
	33 HF-PEF		HF-PEF: 68 (15)		Stable on medication ≥4 weeks		NYHA		3% of the HF-PEF cohort showed evidence of CI
					HF-PEF based on AHA criteria				
Huijts 2013 [30]	491 HF-REF	Age >60 years and ambulatory outpatients only	77 (8)	Prospective	HF-REF: hospitalization within past year	Co-morbid physical illness	HF-REF: LVEF <45%, NYHA II-IV, NT-proBNP >400 pg/ml	AMT	8% of HF-REF group showed evidence of severe CI (AMT ≤7)
	120 HF-PEF				HF-PEF: NT-proBNP ≥400 pg/ml if pt <75 years or ≥800 pg/ml if pt ≥75 years		HF-PEF: LVEF ≥45%		13% of HF-PEF group showed evidence of severe CI (AMT ≤7)
Kindermann 2012 [31]	20 decompensated HF pts	Decompensated HF: non-consecutive admissions to hospital	Decompensated HF: 60 (16)	Prospective	Decompensated HF: caused by ischaemic or DCM, symptomatic HF for ≥6 months, clinical signs of decompensation, for example, raised JVP	Co-morbid psychiatric, neurological or physical illness. Previous diagnosis of CI	LVEF <45%	Multidomain neuropsychiatric battery	Decompensated HF group scored lower than stable HF group in domains of memory, executive control and processing speed
	20 stable HF pts	Stable HF: outpatients	Stable HF: 61 (17)		Stable HF pts: CHF of ischaemic or DCM, NYHA III-IV, no clinical signs/history of decompensation for ≥3 months		NYHA III/IV		Stable HF group scored lower than the healthy control group in domains of intelligence and episodic memory
	20 healthy controls		Healthy controls: 62 (15)						

AHA, American Heart Association; ALID, adjective list of Janke and Debus; AMT, Abbreviated Mental Test; BNP, brain natriuretic peptide; CAD, coronary artery disease; CHF, congestive heart failure; CI, cognitive impairment; CIMS, complex ideational material subset; CO, cardiac output; CV, cardiovascular; CVLT, California Verbal Learning Test; DCM, dilated cardiomyopathy; DRS, Disability Rating Scale; E/A ratio, ratio of mitral peak velocity of early filling (E) to mitral peak velocity of late filling (A); EF, ejection fraction; HF, heart failure; HF-REF, heart failure-reduced ejection fraction; HF-PEF, heart failure-preserved ejection fraction; iDCM, idiopathic dilated cardiomyopathy; IHD, ischaemic heart disease; JVP, jugular venous pressure; LGT-3, Lern und Gedachtnistest; LV, left ventricular; LVEF, left ventricular ejection fraction; LWHFQ, Living With Heart Failure Questionnaire; MDB, mental deterioration battery; MI, myocardial infarction; MMSE, Mini-Mental State Examination; MRI, magnetic resonance imaging; NT-pro BNP, N-terminal prohormone brain natriuretic peptide; NYHA, New York Heart Association; pts, patients; PCI, percutaneous coronary intervention; PVD, peripheral vascular disease; RBMT, Rivermead Behavioural Memory Test; RCPM, raven coloured progressive matrices; SD, standard deviation; WMS, Weschler Memory Scale.

**Table 2:** **Studies examining cognitive changes over time in the heart failure population**

**Study**	**Sample**	**Population**	**Median age in years (SD)**	**Study methodology**	**Inclusion criteria**	**Exclusion criteria**	**CV measures**	**Cognitive assessment tool used**	**Follow-up period**	**Results**
Karlsson 2005 [32]	146 CHF pts	Age >60 years and outpatients	76 (8)	Prospective	EF <45%	Co-morbid psychiatric, neurological or physical illness. Previous diagnosis of CI	LVEF	MMSE	6 months	12% of HF patients had MMSE scores <24 at baseline
					NYHA II-IV		NYHA			And 4% had MMSE scores <24 at 6 months
Tanne 2005 [33]	20 CHF underwent exercise programme 5 CHF pts as control pts	Outpatients	63 (13)	Prospective	EF ≤35%	Co-morbid psychiatric, neurological or physical illness	LVEF	Multidomain neuropsychiatric battery	18 weeks	Improvement in executive function post-exercise programme
					NYHA III		NYHA			
					History of HF for ≥6 months		Mod-Bruce ETT			No change in cognition in control group with time
					Stable on medication ≥6 weeks		6 minute walk test			
Stanek 2009 [19]	40 HF pts, 35 CAD controls	Age >53 and <84 years. Outpatients	70 (8)	Prospective	HF: English speaking	Co-morbid psychiatric or neurological illness. Previous diagnosis of CI	NYHA	DRS	12 months	HF patients improved at 12 months, particularly in attention
					NYHA II or III					
					CO <4 L/minute		CO			Cardiac controls stable at 12 months
					CAD controls: CO ≥4 L/minute, history of MI/CAD					
Almeida 2013 [34]	77 HF pts	Age >45 years and outpatients	HF: 68 (10)	Prospective	HF: EF <40%, English speaking	Co-morbid psychiatric or neurological illness. Previous diagnosis of CI	NYHA	Multidomain neuropsychiatric battery	2 years	CHF group showed cognitive decline compared with CAD and healthy controls
	73 CAD controls		CAD controls: 68 (10)		CAD controls: previous MI and EF >60%, English speaking		LVEF			
	81 healthy controls		Healthy controls: 69 (11)		Healthy controls: no history of CAD, English speaking		6 minute walk test			
Hjelm 2011 [35]	95 HF pts 607 non-CHF controls	Age >80 years and outpatients	84 (3)	Prospective	Not specified	Not specified	HF diagnosis based on documentation in medical records	Multidomain neuropsychiatric battery	10 years	HF patients showed significant decline in episodic memory and spatial performance compared with controls
Riegel 2012 [36]	279 consecutive HF pts (HF-REF and HF-PEF)	Age <80 years and outpatients	62 (12)	Prospective	Stage C HF and English speaking	Co-morbid psychiatric or physical illness. Previous diagnosis of CI	NYHA I-IV	Multidomain neuropsychiatric battery	6 months	No significant change in cognition over 6 months (HF-REF and HF-PEF)
							LVEF			Minimal improvement in DSST in both groups (likely due to learned effect)
										Higher LVEF associated with lower DSST score
Huijts 2013 [30]	491 HF-REF 120 HF-PEF	Age >60 years and outpatients	77 (8)	Prospective	HF-REF: hospitalization within past year	Co-morbid physical illness	HF-REF: LVEF <45%, NYHA II-IV, NT-proBNP >400 pg/ml	AMT	18 months	HF-REF: 23% of HF pts showed decline of ≥1 point in AMT over 18 months
					HF-PEF: NT-proBNP ≥400 pg/ml if pt <75 years or ≥800 pg/ml if pt ≥75 years		120 HF-PEF: LVEF ≥45%			HF-PEF: 24% of HF pts showed improvement of ≥1 point in AMT over 18 months

AMT, Abbreviated Mental Test; CAD, coronary artery disease; CHF, congestive heart failure; CI, cognitive impairment; CO, cardiac output; CV, cardiovascular; DRS, Disability Rating Scale; DSST, digit symbol substitution test; EF, ejection fraction; ETT, exercise tolerance test; HF, heart failure; HF-REF, heart failure-reduced ejection fraction; HF-PEF, heart failure-preserved ejection fraction; LVEF, left ventricular ejection fraction; MI, myocardial infarction; MMSE, Mini-Mental State Examination; NT-pro BNP, N-terminal prohormone brain natriuretic peptide; NYHA, New York Heart Association; pts, patients; SD, standard deviation.

**Table 3:** **Studies examining the relationship between cognitive impairment and outcomes in patients with heart failure**

**Study**	**Sample**	**Population**	**Median age in years (SD)**	**Study methodology**	**Inclusion criteria**	**Exclusion criteria**	**Measures**	**Results**
Zuccalà 2003 [37]	1511 HF pts 11,790 controls	All geriatric or general medical admissions	79 (9)	Prospective	Not specified	Not specified	Hodkinson abbreviated mental test	Mean length of hospital stay: pts with CI = 15 ± 10 days; pts without CI = 15 ± 9 days
							Length of hospital stay	Inpatient mortality: pts with CI, 18%; pts without CI, 3%
							1 year mortality	1-year mortality: pts with CI, 27%; pts without CI, 15%
Karlsson 2005 [32]	146 CHF pts	Age >60 years and outpatients	76 (8)	Prospective	LVEF <45%	Co-morbid psychiatric, neurological or physical illness. Previous diagnosis of CI	HF self-care	Self-care scores were significantly higher in those with MMSE >24 compared to those ≤24
					NYHA II–IV		questionnaire	
							MMSE	
Riegel 2007 [38]	29 CHF pts	Outpatients	64 (10)	Cross-sectional	LVSD on echo	Co-morbid psychiatric or physical illness. Previous diagnosis of CI	Self-care of HF index	CI was worse in the poor self-care group compared to the good and expert self-care groups but did not reach level of significance
					Clinical HF		DSST	
					English speaking		Probed memory recall	
Cameron 2009 [39]	50 CHF pts	Age >45 years and consecutive hospital admissions	73 (11)	Cross-sectional	Clinical CHF	Co-morbid neurological illness. Previous diagnosis of CI	Self-care of HF index	CI was not a predictor of self-care
					LVSD on echo		Cardiac depression scale	
					English speaking		MMSE	
Cameron 2010 [40]	93 CHF pts	Age >45 years and consecutive hospital admissions	73 (11)	Cross-sectional	Clinical CHF	Co-morbid neurological illness. Previous diagnosis of CI	Self-care HF index	CI and self-care management were significantly associated (t = 2.7; *P* < 0.01)
					LVSD on echo		MMSE	
					English speaking		MoCA	
Pulignano 2010 [41]	93 CHF pts	Consecutive outpatients	77 (6)	Cross-sectional	Not specified	Not specified	The European heart failure self-care behaviour scale	MMSE was negatively correlated with self-care behavioural scores (r = 0.58, *P* < 0.001)
							MMSE	
Alosco 2013 [42]	110 CHF pts	Age >50 years and <85 years. Outpatients	70 (9)	Prospective	NYHA II-IV	Co-morbid psychiatric, neurological or physical illness. Previous diagnosis of CI	Lawton-Brody instrumental activities of daily living	Poorer performance on 3MS was associated with worse total activities of daily living performance
					English speaking		Modified MMSE (3MS)	
Harkness 2013 [43]	100 CHF pts	Age >55 years and outpatients	72 (10)	Cross-sectional	Confirmed HF using the Boston criteria	Co-morbid psychiatric illness or previous diagnosis of CI	MoCA	MoCA score of <26 was significantly associated with worse self-care management
					LVEF ≤45%		Self-care in HF index	
					Change in symptoms on previous 3 months		Geriatric Depression Scale	
					English speaking			
Alosco 2013 [42]	175 CHF pts	Age >50 years and <85 years. Outpatients	68 (10)	Cross-sectional	NYHA II-IV	Co-morbid psychiatric, neurological or physical illness. Previous diagnosis of CI	Lawton-Brody instrumental activities of daily living	Poorer executive function was independently associated with poorer total activities of daily living performance
					English speaking		Executive function assessed by FAB and LNS	

CHF, congestive heart failure; CI, cognitive impairment; DSST, digit symbol substitution test; FAB, frontal assessment battery; HF, heart failure; LNS, letter number sequencing; LVEF, left ventricular ejection fraction; LVSD, left ventricular systolic dysfunction; MMSE, Mini-Mental State Examination; MoCA, Montreal Cognitive Assessment Tool; NYHA, New York Heart Association; SD, standard deviation.

Studies describing cognitive impairment (CI) in HF-REF have estimated prevalence at anywhere between 30 and 80% of patients (Table 1). This heterogeneity results from differences in study designs, case mix and cognitive assessments employed. Accepting the limitations of the evidence, even at the more conservative estimates of prevalence, the literature would suggest that CI frequently co-exists with HF-REF (Table 1).

Cross-sectional studies of cognition in HF have value in quantifying the burden of prevalent disease but give no clues as to temporal relationship or causation. To describe the incidence and 'natural history' of cognition in HF ideally requires prospective follow-up of a cohort free from CI at inception. Few studies have utilised this design and, where data are available, the validity is limited by small sample sizes, limited follow-up with substantial attrition and use of cognitive assessment tools that may not be sensitive to modest but clinically meaningful change (Table 2). Inherent in this study design is the assumption that CI follows or is a consequence of the HF pathology [16]. A literature around 'reverse causation' in heart disease has been described. In brief, early studies describing association of psychological or 'personality' factors and heart disease assumed that the neuropsychological traits pre-dated and were probably causative in the development of the cardiac condition. Subsequent data have questioned this temporality and suggest that subclinical (undiagnosed) vascular disease may cause psychological distress phenotypes [44]. Such arguments may also hold for HF and neuropsychological disease, where both cognitive change and psychological distress may be the cause or effect of HF. Investigating reverse causation is challenging but possible; to avoid biases from early mortality, large datasets with sufficient prospective follow-up are required [44].

Association does not imply causation and we must be mindful that both HF and CI are diseases of older age with many shared pathologies. Recognising this, many HF studies have defined an age-related inclusion criterion. With all the caveats that come with the heterogeneity of the available data, it would seem that association of CI and HF is present at all ages (Table 1). Studies that have attempted more sophisticated adjustment for confounders illustrate the inherent difficulty in teasing out what is contributory to cognitive decline and what is association or epi-phenomenon. In general, HF patients tend to have poorer scores on cognitive tests when compared with a 'healthy' (no cardiac disease) control group [34], but this comparator is still potentially confounded by cardiovascular comorbidity in the HF group. Inclusion of a cohort with common vascular risk factors but no HF may allow determination of whether HF *per se* is associated with CI. Where attempts have been made to utilise this design, studies have been modest in size and results contradictory [16,19]. Some authors have described little difference between groups and others have described increased rates of CI in HF-REF groups, particularly in 'executive function' domains.

A direct 'dose response' relationship between severity of HF and severity of CI would strengthen arguments for a causal link. HF-REF can be quantified in terms of EF or symptom burden. For both measures there is an independent association with increasing prevalence of CI [6,8,13,16,17,20] and the poorest scores on cognitive testing are most often seen in those with the severest disease [23]. Interestingly, an association with CI is also seen in those with echocardiographic evidence of reduced EF but without symptoms of HF (that is, patients with asymptomatic left ventricular systolic dysfunction) [7].

Few studies have described cognitive function in patients with HF-PEF [10,22,23,29,30,45], but the pattern seems to be that CI is a substantial problem in all HF regardless of EF. Whether the prevalence or phenotype of cognitive change differs between HF-PEF and HF-REF is not clear as there have been few comparative studies. In keeping with much of the HF and cognition literature, where data are available, there is substantial potential for bias and results are contradictory. Some authors have described higher proportion of cognitive problems in HF-REF [29], while secondary analyses of clinical trials have suggested either an equal proportion of CI across the groups or an excess of CI in those with HF-PEF [30,45].

## Heart failure and delirium

Two patterns of cognitive problems in HF are recognised: a chronic, progressive decline in cognitive ability and a more acute change in cognition often in association with decompensated disease. The acute delirium and HF relationship has not been well described. Delirium is a common sequela of decompensated HF; one study estimated that 17% of unscheduled HF hospitalisations had features of delirium [46]. Where delirium accompanies HF, outcomes are generally poor with increased mortality and length of stay [46]. However, delirium is a frequent complication of most medical emergencies in older adults and the delirium of decompensated HF may be no more or less frequent than the delirium that accompanies other medical conditions such as stroke or pneumonia.

## Impact of cognitive impairment in heart failure

There is a literature describing the relationship between CI and 'classical cardiovascular trial' outcomes (Table 3). In general the presence of CI in HF is associated with poorer clinical outcomes, including longer hospital admissions, increased inpatient mortality and increased 1-year mortality [37]. However, as CI seems to be associated with more severe HF and with other medical comorbidities, we should not assume that poorer outcomes are directly attributable to the cognitive state. Several other important metrics have been described in HF cohorts and all seem to be worsened by the presence of CI, including functional ability, medication adherence and institutionalisation (Table 3). Cognitive decline tends not to occur in isolation and, as with other diseases of older age, the presence of impaired cognition in HF is often associated with concomitant functional decline and poor levels of self-care [32,37,38,40-43,47].

## Potential pathophysiological explanations of cognitive impairment in heart failure

Historically, research describing the pathology of the dementias has been polarised, with vocal proponents for 'amyloid' and 'cerebral small vessel disease' aetiologies. Increasingly these processes are recognised as co-existing with complex biological interactions [48]. The same is likely true of the pathogenesis of CI in HF. Chronic cerebral hypoperfusion and occult cardioembolic disease are exemplar mechanistic explanations that have dominated the literature on cognition in HF. Both processes have face validity, have strong supporting scientific and observational data and yet have traditionally been studied in isolation [49]. For ease of understanding, we will keep this dichotomy and discuss the potential pathological mechanisms separately; however, it seems likely that both processes frequently coexist in patients with HF and may exert pathological synergy.

Although most of the postulated mechanisms we will discuss have been described in the context of HF-REF, issues of cerebral hypoperfusion, thrombotic disease and concomitant cardiovascular disease are also seen in HF-PEF [2] and it seems likely they will factor in the pathogenesis of any cognitive decline seen in this syndrome.

### Confounding from other diseases

Co-existence of dementia and CI has been reported in a variety of cardiovascular disorders, including CAD, myocardial infarction and valvular heart disease. Midlife exposure to the common vascular risk factors of diabetes, hypertension and smoking is associated with later life cognitive decline [16]. This background is relevant to the study of patients with HF as many have a history of one or more of these co-morbidities. As discussed previously, dissecting the contribution of HF from concomitant vascular risk and disease is challenging but is essential for future studies that wish to describe the cognitive component of HF.

AF is a potential confounding condition worthy of separate discussion. The association of AF with cognitive decline is compelling [50]. Much of the CI associated with AF will be driven by cardioembolic stroke. However, cognitive decline is also seen in patients with AF and no history of clinical stroke, possibly representing occult embolic disease [50]. AF is common in HF and prevalence increases with severity of disease. Up to 50% of patients with 'end-stage' HF have AF [51]. Increasing use of ambulatory monitors is discovering substantial undetected paroxysmal AF and so these figures may be underestimates. While AF will be a factor in the pathogenesis of some HF-related CI, it is probably not the sole explanation. Where studies have controlled for the presence of AF in their HF patient population, there remains substantial prevalent CI [10,11,13,16,31].

Any discussion of cognition in cardiac disease has to consider the effect of invasive and instrumental procedures. The interventional toolkit available to cardiologists is increasingly sophisticated, with new indications emerging. Acute and chronic neurological deficits associated with cardiac surgery are well described [52] while interventions such as cardiac catheterisation and transcatheter aortic valve replacement have also been associated with post-procedure CI [53]. The mechanism of neurological insult associated with these procedures is likely a combination of reduced cerebral perfusion and embolic disease.

As well as 'physical' conditions, mood disorder may also represent an important confounder of association between HF and CI. Clinically important depression and anxiety are common in patients with HF. Depression is found in nearly 30% of HF patients and is associated with poor outcomes [54]. There is a complex interplay between cognitive decline (particularly in the context of 'small vessel disease'), mood disorder and systemic vascular disease that is poorly understood but likely to be relevant to HF. Mood disorders are particularly important to detect as they can respond to intervention, making mood disorder in HF a potentially treatable form of cognitive decline.

### Shared pathophysiology (systemic inflammation and amyloid)

Several recent studies have demonstrated the formation of tangle and plaque-like structures and fibrillar deposits (that is, the 'hallmark' lesions of Alzheimer’s disease (AD) dementia) within the myocardium of patients with hypertrophic cardiomyopathy and idiopathic dilated cardiomyopathy [55]. Mis-folded proteins in the form of intermediate oligomers have also been described in cardiac tissue, with a distribution similar to that observed in the brain of patients with AD [55], raising the possibility of a common myocardial and cerebral pathology in a subset of patients with HF.

The systemic inflammatory state recognised in patients with HF may also contribute to CI [56]. It is postulated that inflammatory mediators influence cognition via diverse cytokine-mediated interactions between neurons and glial cells. *In vitro* and animal models support the inflammation and cognitive decline hypothesis and studies in humans with HF are emerging, although data are far from definitive at present [56].

### Acute and chronic hypoperfusion

A mechanistic link between hypotension and CI, mediated via chronic cerebral hypoperfusion and loss of the normal autoregulation of cerebral perfusion pressures, has been postulated. Many diseases, including diabetes mellitus and depression, are associated with impaired reactivity of cerebrovascular perfusion autoregulatory systems and this state seems to confer a higher risk of cognitive decline [57]. HF patients often have systemic hypotension and in the context of disordered autoregulation this could lead to further insults to cerebral perfusion. Cerebral perfusion abnormalities have been demonstrated in HF patients, with reactivity more impaired in patients with greater severity of HF.

These hypoperfusion cognitive problems are not necessarily 'vascular' dementia. In animal models, reduced cerebral blood flow triggers a neurotoxic cascade that culminates in accumulation of amyloid and hyperphosphorylated tau proteins, the classical precursors of AD. If chronic hypoperfusion is causative, then improving cerebral blood flow should reduce cognitive decline. There is some evidence to support this view in patients with severe HF who have undergone cardiac transplant, pacemaker or cardiac resynchronisation therapy, and in whom measures of cognition have stabilised or improved post-procedure [58].

### Thrombosis and cerebral infarction

**Figure 2:**
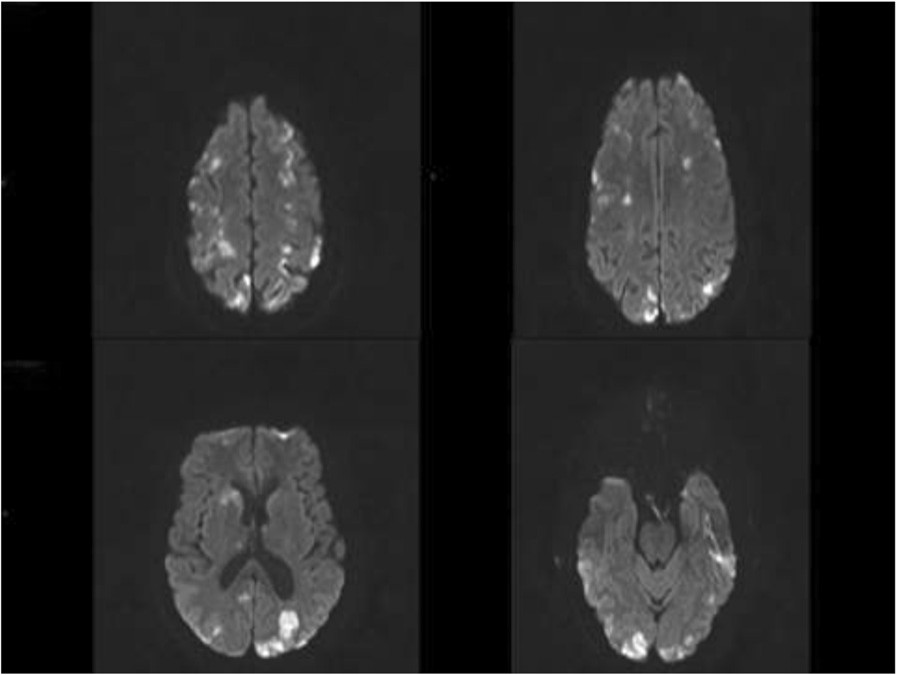
**Magnetic resonance imaging of brain (diffusion weighted imaging sequences) in a patient with severe left ventricular systolic dysfunction and acute cognitive change.** The initial images were felt to represent a multi-infarct state, presumed cardioembolic and 'watershed' (hypoperfusion) infarction. Subsequent investigations revealed that the patient had 'shared' cardiac and cerebral pathology caused by a systemic and cerebral vasculitic process.

The potential importance of AF-related cardioembolism has been discussed. Cardioembolism is also seen in HF with sinus rhythm where ventricular function is the most important determinant of thrombus formation and potential embolic cerebral infarction [59] (Figure 2). Downregulation of thrombomodulin, structural changes in the cardiac chambers and potential blood stasis in the context of reduced myocardial contractility are associated with thrombus formation that may in turn lead to arterial events of clinical stroke or occult cerebral infarction [59]. This systemic prothrombotic phenotype increases risk of all thrombo-embolic diseases and HF is also associated with venous thromboembolism [60,61]. This is not surprising, as abnormalities in all three constituents of Virchow’s Triad (abnormal blood constituents, abnormal vessel wall and abnormal blood flow) are present in HF. Neurohormonal activation seen in HF is associated with increased production of thrombogenic factors such as von Willebrand factor, thromboxane A2 and endothelin. The end result is a hypercoagulable state with increased serum levels of circulating fibrinogen, fibrinopeptide A and D-dimer (amongst others) resulting in platelet and thrombin activation and ultimately leading to plasma hyperviscosity and thrombosis [1]. A relationship between all these circulating markers of thrombosis and haemostasis and cognitive decline, particularly 'vascular dementia', has been described [62]. It would seem intuitive that anticoagulation may prevent sequelae of thrombosis; however, studies of formal anticoagulation in HF with sinus rhythm have been equivocal. To date, no large study of anticoagulation in HF describing cognitive outcomes has been published.

## Cognitive screening in heart failure services

Given the prevalence and potential impact of CI in HF, a case could be made for routine cognitive screening of HF patients. This is a controversial area with strongly held views on both sides. Recent observational data suggest that informal assessment of cognition by a cardiologist is insufficiently sensitive, with around three in four HF patients with important cognitive problems not recognised as such in routine consultations [63]. To date, routine screening for CI has not been incorporated into HF clinical guidelines; this may be due in part to the lack of a standardised screening technique that is feasible and acceptable for use in the cardiology outpatient setting. A recent systematic review of cognitive screening questionnaires utilised in HF studies concluded that the accuracy of traditional cognitive assessment measures is questionable in HF populations and appropriate thresholds/normative values need to be established [64]. In this regard we welcome ongoing work by the Cochrane Dementia and Cognitive Improvement Group to offer synthesis of test accuracy of cognitive assessments in various healthcare contexts [65].

## Treatment implications of cognitive impairment in heart failure

There is an impressive evidence base to support pharmacological interventions in HF-REF. Historically HF trials have described clinical outcomes such as death, vascular events and hospitalisation with decompensated HF. There has been little focus on cognition or dementia as trial outcome or as a case mix adjuster. In fact for many of the trials that inform the HF evidence base, dementia or CI will have been an exclusion criterion. Where trialists have attempted to describe cognitive effects of HF treatment, results have been neutral [30].

Central to the treatment of HF is relatively complex multi-drug pharmacological treatment with attendant need for careful biochemical surveillance and self- monitoring. To achieve optimal outcomes requires strict adherence to prescribed evidence-based therapy [2]. Poor adherence is linked to an elevated risk of hospitalisation and death, whereas appropriate self-management may reduce these risks [2]. It seems intuitive that ensuring adherence and self-management would be especially challenging in the context of CI.

Interventions with angiotensin converting enzyme inhibitors (ACE-is), which have effects on the renin-angiotensin-aldosterone system (RAAS), have been a mainstay of HF-REF therapy for decades. ACE is also important in neurotransmitter modulation and there are theoretical reasons to believe that ACE-is may have an effect on cognitive decline. Cognitive sub-studies of the Cardiovascular Health Study and the Italian Longitudinal Study on Ageing [66,67] both reported that subjects treated with ACE-is had equivalent rates of incident dementia compared with those treated with other antihypertensives. However, there were intriguing within-class differences in cognitive outcomes - for example, between centrally and non-centrally active agents and between differing drug potencies [67]. The other pillars of HF-REF therapy, beta-blockers and mineralocorticoid receptor antagonists, may also influence cognition. Although no studies specific to HF are available, there is hypertension literature suggesting theoretical cognitive effects of beta-blockade but inconclusive evidence that this is clinically important [68]. Cognitive effects of mineralocorticoid receptor antagonists have been demonstrated in animal models but human data are limited [69].

Novel approaches to pharmacological intervention in HF are being developed, with the natriuretic peptide system a key therapeutic target. These peptides possess differing degrees of haemodynamic, neurohormonal, renal and cardiac effects which may be favourable in the HF setting and may augment the effects of RAAS blockade. Preliminary studies using inhibitors of neprilysin (also known as neutral endopeptidase), an enzyme involved in the breakdown of endogenous natriuretic peptides, have yielded encouraging results [70]. Based on this experience a phase III trial comparing the angiotensin receptor neprilysin inhibitor molecule LCZ696 to the ACE-i enalapril was undertaken in chronic HF-REF (PARADIGM-HF). This trial was recently stopped for benefit of LCZ696 over enalapril [71]. However, cardiac optimism must be tempered by caution regarding potential non-cardiac, cognitive adverse effects. Mutations in the neprilysin gene have been associated with familial forms of AD and neprilysin-deficient mice show an AD phenotype [72].

In the light of non-definitive data, how should we treat a patient with HF and CI? Cognitive enhancing medication such as acetylcholinesterase inhibitors have recognised effects on the cardiac conduction system, occasionally causing bradycardia, sick sinus syndrome or other arrhythmias (including torsades de pointes) resulting from QT prolongation through excessive cholinergic stimulation. One recent study showed donepezil to be safe in patients without symptomatic heart disease and actually reduced levels of plasma brain natriuretic peptide in patients with subclinical HF [73].

Although there are no data to suggest cognitive benefits of standard HF therapy, there are equally no signals of harm. Given the beneficial effects of pharmacological therapy on mortality and hospitalisation, it would seem sensible to consider these evidence-based medical interventions for all HF patients, tailoring the intervention to suit the patient. A multidisciplinary approach with frequent review and medication titration seems to work well. Prescribers need to be alert to the potential effects of CI on concordance with sometimes complex drug regimens. Early use of compliance aids and involvement of family or carers may help in this regard. The goal of management of HF is to provide 'seamless care' in both the community and hospital to ensure the treatment of every patient is optimal. Despite the plethora of publications and guidelines, the data consistently show a lower uptake of evidence-based investigations and therapies in older patients with consequent higher rates of HF hospitalizations and mortality [43]. The current shift away from concentration on individual drug therapies to a focus on systems of care that allow effective treatment delivery is welcomed.

## Conclusion

Recurrent themes in our synthesis of the literature regarding CI and HF are a lack of primary data, methodological limitations in available research, and conflicting results. To progress our understanding we recommend increasing use of cognitive assessment using standardised screening tools in all future HF studies. Although we found numerous studies assessing prevalence, there is a dearth of studies investigating the incidence of CI in HF. Once the incidence and prevalence of CI in HF are better defined we need to evaluate the consequences of CI in HF. Identifying underlying mechanisms for CI in HF may present targets for intervention, the 'holy grail' of cognitive research. A number of processes have been postulated, and we now need confirmatory studies using new developments in neuroimaging and biomarkers in representative populations of HF patients. All of this will require a multidisciplinary approach between HF and dementia research teams. Such collaborative activity is urgently needed given the projected increases in both CI and HF.

## Note

This article is part of a series on *The impact of acute and chronic medical disorders on accelerated cognitive decline’*, edited by Carol Brayne and Daniel Davis. Other articles in this series can be found at http://alres.com/series/medicaldisorders.

## Abbreviations

ACE-(i): Angiotensin converting enzyme-(inhibitor)
AD: Alzheimer’s disease
AF: Atrial fibrillation
CAD: Coronary artery disease
CI: Cognitive impairment
EF: Ejection fraction
HF: Heart failure
HF-PEF: Heart failure-preserved ejection fraction
HF-REF: Heart failure-reduced ejection fraction
RAAS: Renin-angiotensin-aldosterone system.
